# Artificial intelligence in personalized computed tomography dose optimization: a systematic review of techniques and clinical outcomes

**DOI:** 10.3389/fmed.2026.1791498

**Published:** 2026-09-03

**Authors:** Nora Almuqbil

**Affiliations:** Department of Radiological Sciences, College of Health and Rehabilitation Sciences, Princess Nourah bint Abdulrahman University, Riyadh, Saudi Arabia

**Keywords:** artificial intelligence, computed tomography, image quality, personalized dose, radiation dose reduction

## Abstract

**Introduction:**

Artificial intelligence (AI) for personalized computed tomography (CT) dose optimization has gained increasing attention due to its potential to reduce radiation exposure while maintaining image quality. CT dose optimization tailors according to individual patient characteristics, including age, weight, body composition, and clinical condition. The study aims to evaluate AI techniques in CT dose optimization, focusing on reducing radiation exposure while maintaining image quality.

**Methods:**

The relevant studies published between 2010 and 2024 were screened from databases, such as PubMed, IEEE Xplore, Scopus, ScienceDirect, and Web of Science, by following “Preferred Reporting Items for Systematic reviews and Meta-Analyses (PRISMA)” guidelines and “Population, Intervention, Comparison, and Outcome (**PICO**)” model. Risk of bias analysis revealed possible biases in studies. Meta-analysis was performed to elucidate the symmetry of the evidence and data from relevant studies.

**Results:**

The review underscores the significance of deep learning (DL) and machine learning (ML) algorithms, including reconstructive strategies, predictive modeling, and hybrid models, as critical in enhancing dose optimization.

**Conclusion:**

AI-driven approaches in CT dose optimization show promising potential to reduce radiation exposure safely and effectively without compromising image quality. Future research necessitating larger, diverse clinical trials are essential to validate these findings and ensure the equitable application of AI technologies across varied patient demographics. Studies have consistently indicated AI’s potential to reduce the dose exposure while maintaining the image and diagnostic quality. They highlight the importance of DL- and ML-based algorithms, such as reconstructive strategies, predictive modeling, and hybrid models, in CT dose optimization.

## 1 Introduction

High-resolution technical imaging modalities, such as computed tomography (CT), magnetic resonance imaging (MRI), ultrasounds, positron emission tomography (PET), nuclear medicine imaging, and X-ray, excel at providing details regarding lesion location, size, morphology, chemical composition, and structural changes to adjacent tissues (1, 2), joint abnormalities or injuries, spinal injuries, etc. With the advancement of technology, the field of medicine has revolutionized. Among the above-mentioned imaging modalities, CT is one of the sought-after tools for diagnosing different diseases, providing detailed cross-sectional images of a body, thus aiding in treatment plans. Nevertheless, patients getting exposed to radiation from CT scans, particularly children and other highly sensitive populations, have been an increasing concern. Radiation exposure can lead to the deposition of a certain amount of matter in the body, thus triggering cell damage or cancer, which is fatal and detrimental to human health. Thus, developing numerous methods for optimizing CT doses to reduce radiation exposure is imperative. In recent years, numerous strategies have been developed to decrease the dose while not compromising image quality (3, 4).

Conventional techniques focus on adjusting parameters such as tube current, voltage, and scan time but are often based on fixed protocols (5). Therefore, to develop new methods and protocols to reduce the CT dose, it is necessary to understand the relationship between image quality and radiation dose (6).

For example, a study indicated that there are several parameters to quantify the CT scan image quality, such as Contrast-to-noise ratio (CNR) and signal-to-noise ratio (SNR) (7). CNR is the intensity difference between the tissue in question and the surrounding tissue, while SNR is the signal strength of the image to the background noise. High-contrast spatial resolution, both in-plane and cross-plane (along the z-axis), quantifies the minimum size of high-contrast objects that can be resolved. Low-contrast spatial resolution quantifies the minimum size of low-contrast objects that can be differentiated from the background, which is related to the material’s contrast and the system’s noise-resolution properties.

Numerous traditional strategies follow the ALARA principle to reduce the dose exposure, such as manually reducing the tube current and voltage for pediatric or adult patients as per their weight or body size, using alternative modalities (MRI or ultrasound) where appropriate, clearly defining the area of interest to be scanned to prevent irradiating regions in the body that are not relevant, using beam collimator to minimize X-ray exposure to adjacent tissue, employing external lead shielding to protect organs, like breast, thyroid, etc., that are radiosensitive, double-checking the scan parameters to avoid any errors or repeated scans, regularly maintain CT scanners for optimal performance (8, 9).

Artificial intelligence has been at the forefront of technological innovation, reshaping medical science and the healthcare industry. It uses learning algorithms and methods such as deep learning and machine learning to detect diseases, enhancing data-driven patient care (10). These learning tools study patient data to understand radiation sensitivity and improve the accuracy and precision of dose optimization through automation by considering a wide range of patient-specific variables. ML models, reconstruction-based algorithms, and hybrid approaches ensure patient safety while maintaining the high diagnostic standards imperative to modern medical care. The ability of AI to analyze imaging requirements and adjust exposure parameters dynamically ensures an optimal balance between image quality and minimal radiation.

Computed tomography dose optimization focuses only on a handful of patient-specific parameters, including age, BMI, and clinical history. However, a streamlined approach would integrate large data sources such as electronic health records (EHRs), genetic data, and imaging data. However, this integration brings challenges such as data heterogeneity and privacy breaches (11). The study aims to evaluate AI techniques in CT dose optimization, focusing on reducing radiation exposure while maintaining image quality and identifying research gaps and biases in current literature.

## 2 Methods

### 2.1 Search strategy

For proceeding with this systematic review, the following Population, Intervention, Comparison, and Outcome (PICO) statement was framed to address, clearly define, and kickstart this comprehensive study: For patients (Population) undergoing CT scans across various medical specialities, how do AI-based techniques (Intervention) be used in CT dose optimization. How are those methods better than the traditional methods used for CT dose optimization (Comparison)? What improvements were witnessed in clinical outcomes (Outcome)?

Following the PICO statement that laid the foundation for the study, the scoping and systematic search of relevant studies was conducted through major medical and engineering databases, such as PubMed, IEEE Xplore, Scopus, ScienceDirect, and Web of Science, to identify and map the research landscape of AI in CT dose optimization from 2010 to present. This study started as per the “Preferred Reporting Items for Systematic reviews and Meta-Analyses (PRISMA)” guidelines and PICO model (12). It involved identifying the research question, defining inclusion and exclusion criteria, selecting relevant studies, extracting and collating, summarizing and reporting data, assessing quality, synthesizing and presenting results. Keywords such as radiation dose reduction, radiology, personalized CT dose optimization, AI-driven dose reduction, and patient-specific CT were used to search.

### 2.2 Selection criteria

Moreover, inclusion criteria involved studies using AI algorithms for dose modulation or image quality enhancement, compared radiation dose reduction or image quality improvement outcomes, and focused on clinical applications across different patient demographics. Exclusion criteria included studies that did not use AI for dose optimization or focused on non-human subjects.

### 2.3 Study selection

**FIGURE 1:**
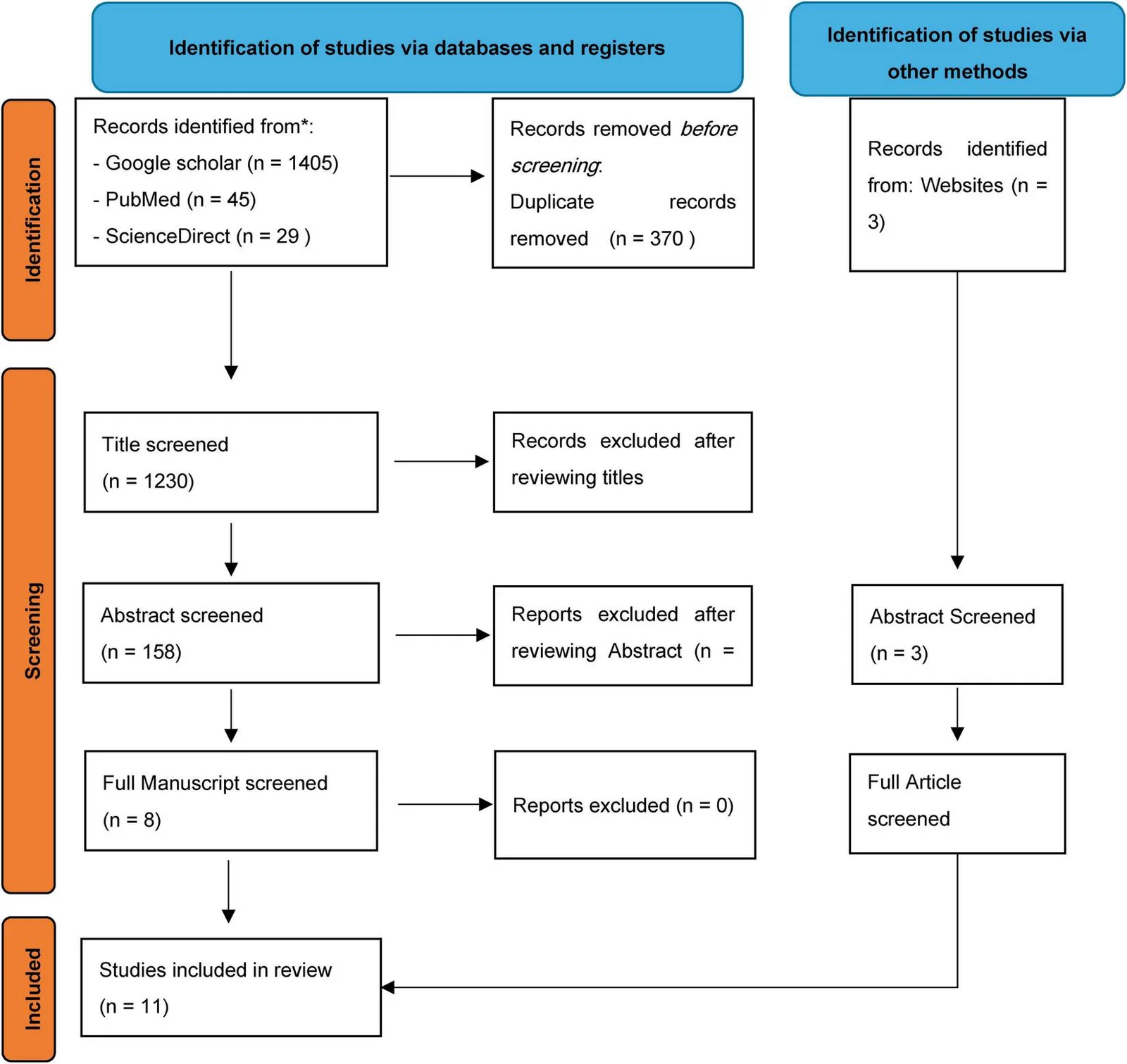
Shows the Preferred Reporting Items for Systematic reviews and Meta-Analyses (PRISMA) flowchart of the study selection process.

Figure 1 illustrates the PRISMA diagram of this scoping and systematic review. It highlights studies from databases and websites, different stages of the selection and screening process, and the number of articles retained and removed after reviewing different sections (abstract and titles) of the studies.

The application of AI-driven CT dose optimization techniques was critically analyzed and understood from various studies. DL models, particularly convolutional neural networks (CNNs) and generative adversarial networks (GANs) have been applied to predict optimal dose settings based on patient data. A study indicates that encoder and decoder CNN demonstrate potential in noise reduction, structural preservation, lesion detection, etc., at a significant pace (13). These models learn from large datasets to correlate patient features (e.g., body size, age, scan type) with the dose required for diagnostic-quality images.

These methods have synthesized different contrast modality images for training networks for multi-modality segmentation, image harmonization, and missing modality synthesis (14). ML algorithms use statistical methods to adjust real-time parameters like tube current and voltage, minimizing radiation exposure without compromising image quality. Reconstruction-based AI can also be applied to the reconstruction phase of CT imaging, where algorithms such as iterative reconstruction (IR) and AI-enhanced IR help improve image quality even when lower doses are used (15). IR has long been used to enhance image quality; however, AI-enhanced IR takes a step ahead through DL algorithms. These models excel at noise reduction, artifact correction, and edge enhancement, compensating for image degradation that typically occurs at lower doses (16–18). Therefore, clinicians can obtain diagnostically impactful images while abiding by the ALARA principle for radiation safety (19). Personalized dosing enhances radiation safety and adheres to the ALARA principle, which is the basis of radiation protection in medical imaging (20).

Considering patient-specific variables, clinical needs, medical history, and anatomical variations, these systems integrate AI algorithms for automated exposure control and scan range limitation. Therefore, hybrid models are one of the best comprehensive and adaptive dose optimization strategies for maintaining image quality while reducing dose exposure. This integration of conventional and AI-driven strategies highlights the potential of personalized dose reduction and imaging protocols in routine clinical practice. These data-driven decisions mark a significant advancement over one-size-fits-all protocols.

## 3 Results and discussion

This systematic and scoping review has identified legitimate studies on AI in CT dose optimization involving patient-centric variables. Studies have consistently indicated AI’s potential to reduce the dose exposure while maintaining the image and diagnostic quality. Most of the studies in this review highlight the use and importance of DL- and ML-based algorithms, such as reconstructive strategies, predictive modeling, and hybrid models, in CT dose optimization.

Artificial intelligence has particularly benefited pediatric CT imaging, where reducing radiation exposure is paramount (21). These techniques have also shown promise for obese patients, where traditional methods may be less effective. AI algorithms read increased body mass, adjusting CT settings to ensure optimal imaging while minimizing radiation exposure. In elderly and high-risk patients, particularly in pregnant females, geriatrics, and oncology, where treatment and repetitive diagnosis are required at every stage of the treatment, AI-driven dose regulation strategies are particularly valuable in reducing radiation exposure without compromising diagnostic efficacy (22).

### 3.1 Challenges in clinical adoption

Large data sets are required to train AI models, enabling them to recognize patterns and provide real-time high-value solutions. However, there are a few drawbacks to adopting AI in clinical practice. AI-driven systems rely on large amounts of data for their training and validation, which raises concerns about patient data privacy, thus hampering clinical safety standards. Protecting patient-sensitive information while ensuring high-level AI training is a crucial challenge. Thus, AI-driven tools must comply with regulatory standards set by the designated authorities to mitigate data leakage risks. Incorporating AI into clinical practice requires sophisticated integration with existing CT scanners and hospital IT systems. However, due to old and outdated infrastructure, numerous hospitals may struggle to integrate AI into existing systems. Moreover, the healthcare industry may hesitate to trust AI-driven technologies without sufficient evidence of reliability, safety, and efficacy. Thus, effective training and educating clinicians on the role of AI in decision-making is imperative for adoption (23).

**FIGURE 2:**
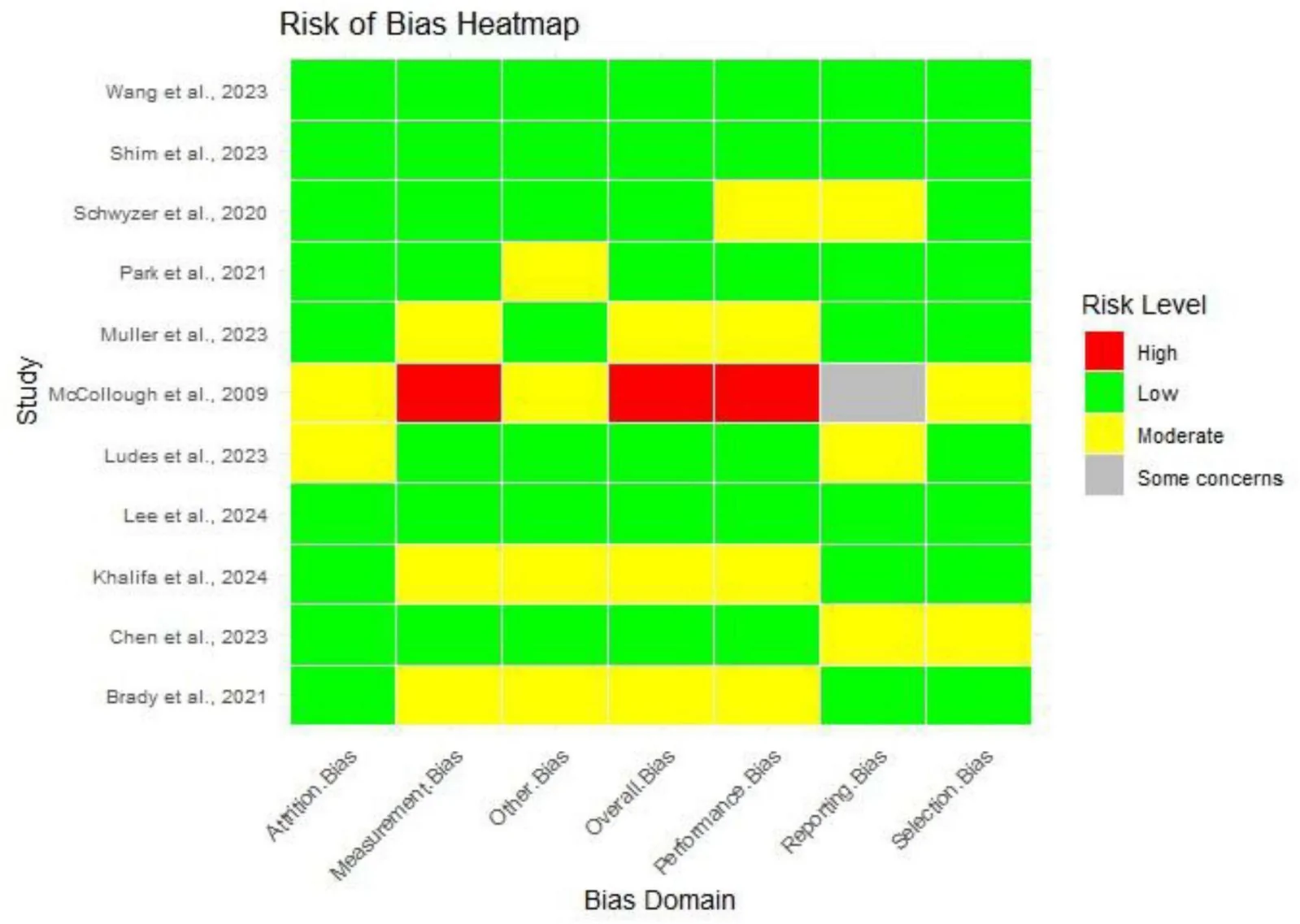
The heatmap shows risk of bias for each study including different bias domains such as attrition bias, measurement bias, other biases, performance bias, reporting bias, and selection bias.

In Figure 2, Green Cells show low risk of bias suggesting that the studies were well-conducted. The yellow cells show that some of them have moderate risk in certain domains, indicating minor methodological concerns, which is common in systematic reviews and should not necessarily disqualify a study. Moreover, red cells, particularly in the study by McCollough et al. (9), show high risk in performance bias and overall bias, indicating serious concerns about its methodology. This study also has a gray cell under selection bias, meaning there are some concerns about how participants were chosen. While most studies show low risk of bias, the presence of some moderate and high-risk studies suggests that methodological differences exist. The high-risk study may be influencing heterogeneity and should be carefully examined in sensitivity analyses. Selection bias and performance bias should be considered while interpreting the outcomes.

**FIGURE 3:**
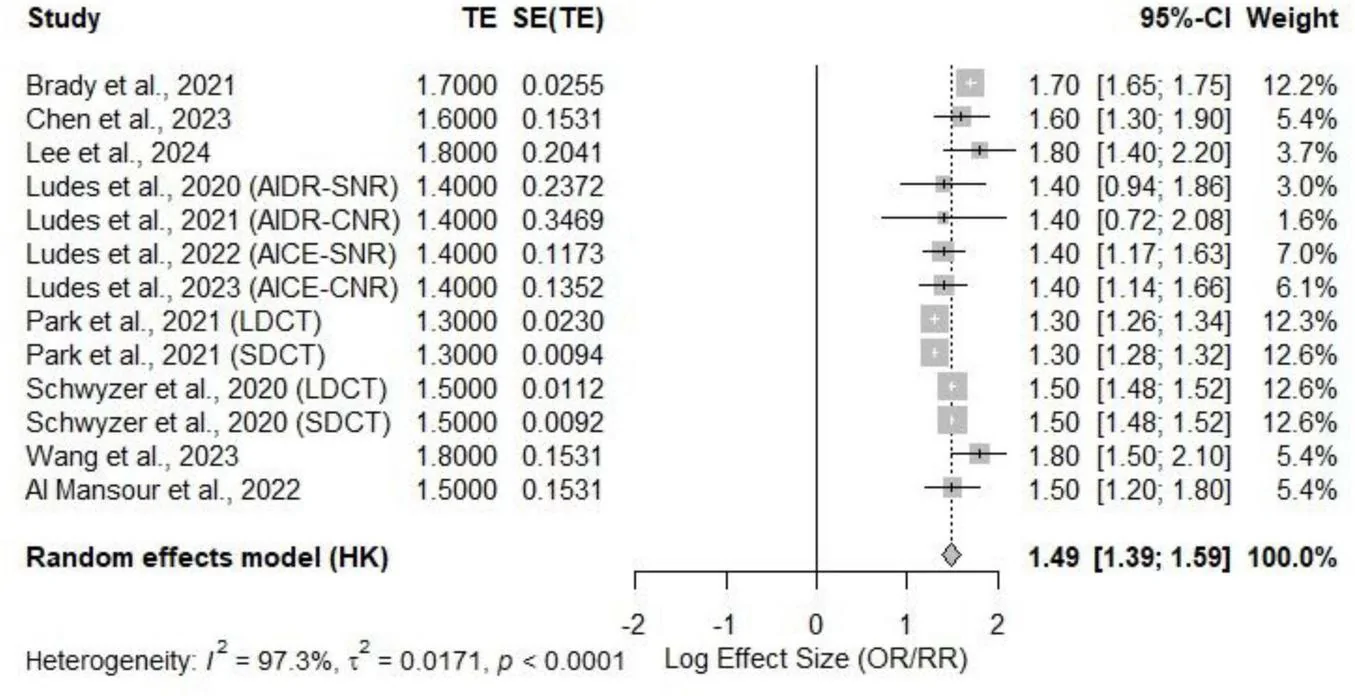
(Effect size analysis) the forest plot visually represents the effect sizes and confidence intervals (CI) of the studies. It also provides a pooled estimate using a random effects model.

The pooled estimate of effect size is 1.49 [1.39, 1.59], indicating statistically significance as the confidence interval does not cross 1 (Figure 3). Heterogeneity is very high (97.3%) showing substantial variability between studies. This suggests that the included studies are quite different in terms of methodology, populations, and interventions. As presented, the *p*-value for heterogeneity is *p* < 0.0001, confirming that the variation is statistically significant. Some studies, such as Brady et al. (24) (12.2%), Park et al. (25) (12.3%, 12.6%), contribute more weight to the analysis, while, other studies, particularly those with larger standard errors, contribute less weight [e.g., (28), 3.7%]. Most studies report effect sizes between 1.3 and 1.8, indicating a generally consistent trend. Some studies show wider confidence intervals due to larger standard errors. The random-effects model accounts for variability across studies and still finds a significant effect. The findings suggest a meaningful effect, but the high heterogeneity calls for cautious interpretation. Differences in study design or populations may be influencing the variability in effect sizes. Thus, subgroup analysis may be necessary to identify the sources of heterogeneity.

A total of 13 studies were included in the meta-analysis. The pooled estimate using a random-effects model (Hartung-Knapp adjustment) yielded a summary effect size of 1.4894 (95% CI: 1.3894 to 1.5893, t = 32.48, *p* < 0.0001). This indicates a statistically significant overall effect. Substantial heterogeneity was observed among the included studies (Q = 441.03, df = 12, *p* < 0.0001). The estimated between-study variance (tau^2^) was 0.0171 (95% CI: 0.0062 to 0.0875), with a tau value of 0.1307 (95% CI: 0.0790 to 0.2957). The I^2^ statistic indicated that 97.3% (95% CI: 96.4% to 98.0%) of the total variability was due to heterogeneity rather than chance, suggesting considerable inconsistency in effect sizes across studies. The H statistic was 6.06 (95% CI: 5.26 to 6.99), further supporting the presence of high heterogeneity.

**FIGURE 4:**
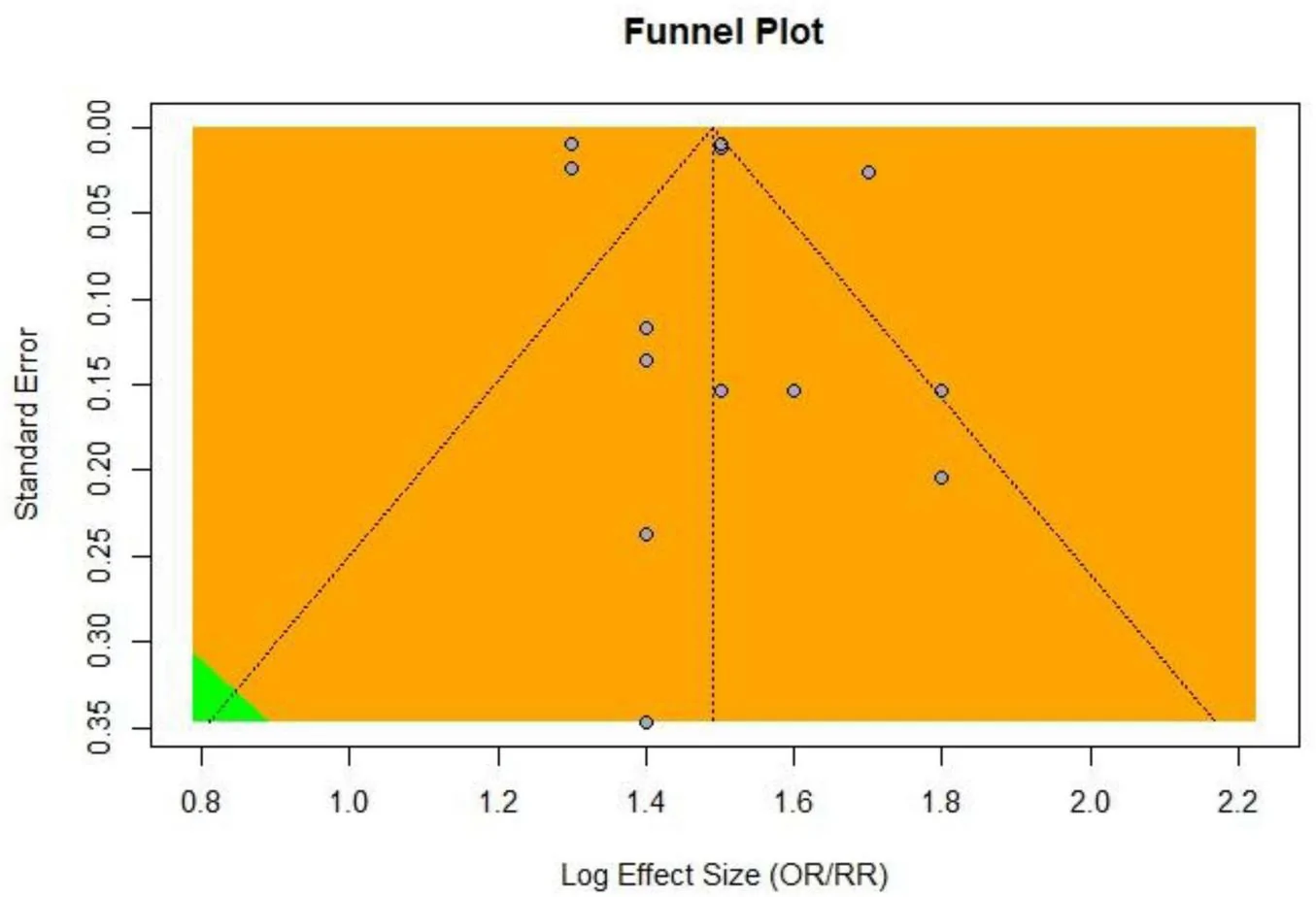
The funnel plot assesses publication bias in meta-analyses.

Assessment of publication bias was conducted using the Egger’s regression test for funnel plot asymmetry (Figure 4). The test result (t = 0.50, df = 11, *p* = 0.6295) did not indicate significant small-study effects. The estimated bias was 1.1291 (SE = 2.2751), suggesting no substantial evidence of publication bias (Figure 4). Individual study effect sizes and confidence intervals are presented in Table 1. The largest weight contributions to the random-effects model were from Park et al. (25), (SDCT) (12.6%), Schwyzer et al. (26), (SDCT) (12.6%), while the smallest contributions came from Ludes et al. (27), AIDR-CNR) (1.6%), Lee et al. (28) (3.7%). Effect sizes ranged from 1.3000 to 1.8000, with most confidence intervals not overlapping 1.0, indicating consistent directionality in the observed effects. These findings suggest a robust and significant overall effect, with high heterogeneity but no indication of publication bias. A linear regression test of funnel plot asymmetry was conducted to assess potential publication bias. The test yielded a t-value of 0.50 with 11 degrees of freedom (df = 11) and a corresponding *p*-value of 0.6295, indicating no significant evidence of asymmetry in the funnel plot. The estimated bias was 1.1291 with a standard error (SE) of 2.2751. Further details of the analysis revealed a multiplicative residual heterogeneity variance (τ^2^) of 39.2155. The predictor variable used was the standard error, with inverse variance weighting applied to the model. This analysis follows the approach outlined by Egger et al. (29) in the BMJ.

**TABLE 1:** A few key findings of artificial intelligence (AI) in personalized computed tomography (CT) dose optimization in various studies.

References	Type of article	Main findings/conclusions	Country of study	Population	Intervention	Outcome
Brady et al. (24)	Original research	Deep learning reconstruction (DLR) improved image quality and dose reduction without sacrificing noise texture and spatial resolution.	USA	Pediatric patients undergoing CT imaging	Deep learning reconstruction (DLR) for CT image enhancement	Improved image quality, greater object detectability, and higher radiologist confidence ratings
Chen et al. (13)	Experimental study	This study utilized a residual encoder-decoder CNN for low-dose CT imaging, improving image quality and preserving diagnostic accuracy at reduced radiation level	China	Low-dose CT imaging applications	RED-CNN for CT image noise reduction	Reduced noise, preserved structural details, and enhanced lesion detection in CT imaging
Khalifa and Albadawy (22)	Systematic review	AI significantly enhances clinical prediction across multiple domains, improving accuracy and efficiency.	Australia	General clinical applications	AI for clinical prediction	Enhanced accuracy and efficiency in clinical prediction
Lee et al. (28)	Prospective study	Low-dose CT using deep learning denoising provides comparable diagnostic performance to standard-dose CT.	South Korea	296 patients with liver tumors	Deep learning-based CT denoising	Comparable diagnostic performance at reduced radiation dose
Ludes et al. (27)	Observational study	Deep learning reconstruction can improve image quality while reducing radiation dose in abdominal MDCT.	France	66 patients undergoing abdominal MDCT	Deep learning reconstruction	Improved image quality with reduced radiation dose
McCollough et al. (9)	Technical review	CT dose optimization strategies help reduce radiation exposure without compromising diagnostic utility.	USA	CT imaging applications	CT dose optimization strategies	Reduced radiation exposure while maintaining diagnostic accuracy
Muller et al. (46)	Preclinical study	Deep learning reduces dose requirements while maintaining high-quality micro-CT images.	Belgium	38 mice in micro-CT	Deep learning-based noise reduction	Lower dose requirements while preserving image quality
Park et al. (25)	Retrospective study	Deep learning denoising in low-dose liver CT improves image quality and maintains diagnostic performance.	South Korea	80 patients with liver lesions	Low-dose CT with deep learning	Non-inferior image quality with deep learning denoising
Schwyzer et al. (26)	AI evaluation study	AI-based pneumonia detection in dose-equivalent CT is feasible but requires protocol optimization.	Switzerland	100 patients undergoing chest CT	AI-based pneumonia detection	Feasible AI-based pneumonia detection, but needs optimization
Shim et al. (47)	Experimental study	Deep learning reduces radiation dose while maintaining image quality in urolithiasis CT protocols.	South Korea	Human phantom study	Deep learning for dose optimization	Dose reduction with maintained image quality
Wang et al. (30)	AI in imaging study	AI-based PET dose reduction strategies achieve high image quality at ultra-low-dose settings.	USA	21 pediatric patients with cancer	AI transformers for dose reduction in PET scans	High-quality PET images with ultra-low-dose AI reconstruction

Research with a higher number of participants [e.g., Brady et al. (24), Wang et al. (30), Trattner et al. (5)] tends to produce more reliable results. These studies incorporate diverse patient populations, making their conclusions more applicable to real-world clinical settings. Well-structured methodologies help minimize bias, ensuring that AI-driven CT dose optimization strategies are tested under realistic conditions. Studies that rely primarily on existing literature [e.g., Kutanzi et al. (31), McCollough et al. (9), Goo (4)] often carry a higher risk of bias due to their dependence on secondary data sources. Without direct patient involvement or new experimental validation, these studies may not fully capture the real-world performance of AI-driven techniques. While they provide useful overviews, their findings should be interpreted cautiously, particularly when assessing the clinical effectiveness of AI applications (32).

Research with fewer than 200 patients [e.g., Histed et al. (1), Khalifa and Albadawy (22)] faces challenges in demonstrating consistent AI performance across different patient demographics. With limited data, AI models are more prone to overfitting, meaning they may perform well in controlled settings but struggle when applied to broader patient populations. Smaller sample sizes also make it difficult to assess whether AI-based dose optimization strategies work equally well across age groups, body types, and medical conditions. Studies that do not disclose their patient sample size or demographics [e.g., Mello-Thoms and Mello (33), Khalifa and Albadawy (22)] introduce uncertainty in their findings. Transparent reporting of patient characteristics is essential for evaluating AI effectiveness. Without clear documentation, it is difficult to determine whether the reported benefits of AI-driven dose optimization apply across a broad patient population or are specific to a narrow subset.

Optimizing radiation doses in CT imaging has become an essential focus of research, especially with the integration of AI technologies that aim to improve image quality while minimizing radiation exposure. This review evaluates 30 studies (spanning experimental research, systematic reviews, and clinical applications) focused on using AI in CT radiology, particularly in dose reduction.

Multiple studies have demonstrated that AI-based approaches can significantly reduce radiation dose (e.g., 20%–40%) compared to conventional fixed-dose protocols, particularly in pediatric populations and patients with low body mass index (BMI) (24). By personalizing the dose parameters to patient-specific characteristics, such as age, medical history, and body composition, AI uses algorithms to cut down unnecessary radiation exposure. This is particularly beneficial for high-risk patients, including children and pregnant women, who require frequent checkups. Studies show that AI technology has been successfully adopted in clinical practice for obtaining high-quality visualization of complex anatomy, such as abdominal, chest, and pediatric CT imaging, without compromising diagnostic accuracy (34). For instance, AI has proven valuable in pediatric imaging regarding radiation safety, substantial dose reduction, and diagnostic accuracy. Such clinical successes highlight the potential of AI in routine radiology practices. In summary, AI has demonstrated an innovative and transformative step forward in health care by adopting personalized imaging protocols and diverse clinical applications. Further adopting advanced protocols and strategies in health care will likely improve patient safety imaging workflows and set new standards in diagnostic radiology.

Brady et al. (24), Nagayama et al. (35) demonstrated that deep learning-based reconstruction enhances image quality in pediatric and adult CT scans, leading to substantial dose reductions while preserving diagnostic accuracy. Research by Zhang and Seeram (15), Chen et al. (13) further confirmed that AI-driven techniques, including denoising and iterative reconstruction, reduce noise and enhance image visualization.

Studies by Costello et al. (8), McCollough et al. (9) identified various dose reduction methods, underscoring AI’s role in adaptive exposure control and automated protocol optimization. Wang et al. (30) demonstrated a 40% dose reduction in whole-body PET-CT scans using AI transformers. As highlighted by Noid et al. (6), Iterative reconstruction algorithms also effectively reduce dose in 4DCT imaging. Ng (21) highlighted AI’s role in pediatric radiology, specifically optimizing radiation exposure while maintaining high-quality images. Kutanzi et al. (31), Demoor-Goldschmidt and de Vathaire (36) emphasized AI’s importance in minimizing radiation risk to children more sensitive to ionizing radiation. Koçak et al. (37), Mello-Thoms and Mello (33) explored challenges in AI, particularly focusing on bias and the need for standardized protocols, recommending robust datasets to ensure generalization. Trattner et al. (5), Yu et al. (7) discussed the significance of protocol standardization and how AI could automate radiation dose adjustments tailored to individual patients.

### 3.2 Limitations and challenges

Variations in scanner models, imaging protocols, and patient demographics impact the performance of AI models (38). Rigorous validation and regulatory approval are required before clinical implementation. AI models require significant computational resources, which may be impractical in some clinical environments. Lack of transparency in AI decision-making remains a barrier for widespread clinical adoption. This review affirms that AI techniques significantly contribute to optimizing radiation doses in CT imaging, enhancing image quality and diagnostic confidence. However, further research is necessary to address challenges such as bias, standardization, and interpretability, with an emphasis on multi-center trials and real-world validation to ensure broad clinical adoption (39). Currently, CT dose optimization focuses on a handful of patient-specific parameters such as age, BMI, and clinical indications. However, a streamlined approach would be to integrate large and varied data sources electronic health records (EHRs), genetic data, and imaging data (40). However, this integration brings with it challenges such as heterogeneity of data and privacy breach (11). The clinical translation of AI-driven CT dose optimization faces regulatory scrutiny across jurisdictions. In the US, AI software used for diagnostic purposes must comply with FDA’s Software as a Medical Device (SaMD) framework. In Europe, CE marking under the MDR is required.

### 3.3 Areas requiring further investigation

High-risk patient groups, including cancer patients, pediatrics with chronic conditions, and pregnant women, suffer from unique challenges during diagnostic imaging compared to other patients. Such groups of patients are highly vulnerable to radiation exposure, making personalized CT dose optimization protocols vital for their safety and care. Cancer patients undergo frequent and repetitive imaging for diagnosis and monitoring therapy response at different stages of the treatment, leading to cumulative high radiation exposure that increases the risk of secondary malignancies. Research should focus on longitudinal dose management, reducing cumulative exposures over time (36). Aligning imaging with therapeutic interventions, such as radiotherapy, could enhance safety and diagnostic efficacy.

Children are susceptible to radiation as their tissues are robust and continuously developing, thus increasing the risk of acquiring lifetime malignancies such as radiation-induced cancers. Such fatal illnesses, such as congenital heart defects or cystic fibrosis, require frequent imaging (31). Therefore, studies should focus on developing age- and size-specific AI-driven reconstruction techniques that reduce noise while maintaining the image quality. Furthermore, AI-based motion-correction technologies, such as during patient movement during a CT scan procedure, could also address challenges related to image quality. Pregnant women require careful imaging for both maternal and fetal health. Therefore, studies should explore AI-driven fetal shielding models to reduce exposure while maintaining safety and diagnostic accuracy for crucial maternal conditions [Yaseen and Rather (41)]. Also, exploring low-dose imaging protocols and integrating non-ionizing modalities could improve safety. Addressing the gaps mentioned above through thorough data collection, training, validation, and compliance with ethical standards will transform the imaging scenario in health care through AI.

Artificial intelligence-driven CT dose optimization, particularly in pediatric and oncology populations, offers potential for reducing the cumulative radiation dose, thereby lowering long-term risks such as radiation-induced malignancies. Longitudinal studies have shown a positive correlation between cumulative radiation exposure and secondary cancer risk. By reducing radiation by 20%–40% (24, 30), AI can contribute to reduced DNA damage and mutagenic potential, ultimately improving long-term safety, especially in vulnerable groups requiring repetitive imaging.

## 4 Conclusion

Artificial intelligence is at the forefront of technological innovation in the healthcare field. It transforms personalized CT dose optimization by reducing radiation exposure while maintaining the image quality. Such advanced and sophisticated techniques can minimize unnecessary radiation while enhancing image quality and patient safety. These potential advancements hold promise, particularly for high-risk populations, such as pediatric patients, oncology patients, and pregnant females requiring frequent imaging. Techniques such as deep learning and machine learning algorithms enable automated personalized adjustments of scanning parameters, including tube current, voltage, and scan duration, to suit patient-specific needs (42, 43). Furthermore, AI-based iterative reconstruction algorithms allow for high-quality imaging even at minimal doses, ensuring that diagnostic standards are met while minimizing exposure (3).

Despite these advancements, a few obstacles hinder the widespread adoption of AI in CT dose optimization. Training and validation of AI through large datasets compromise patient data privacy (2). Therefore, AI models must comply with various regulatory approval standards, given the need for extensive validation and adherence to safety standards. In addition, integrating AI tools into existing clinical workflows poses technical and logistical challenges. Due to outdated infrastructure, numerous healthcare systems cannot support advanced AI models, including real-time data processing capabilities and interoperability with existing imaging systems.

**FIGURE 5:**
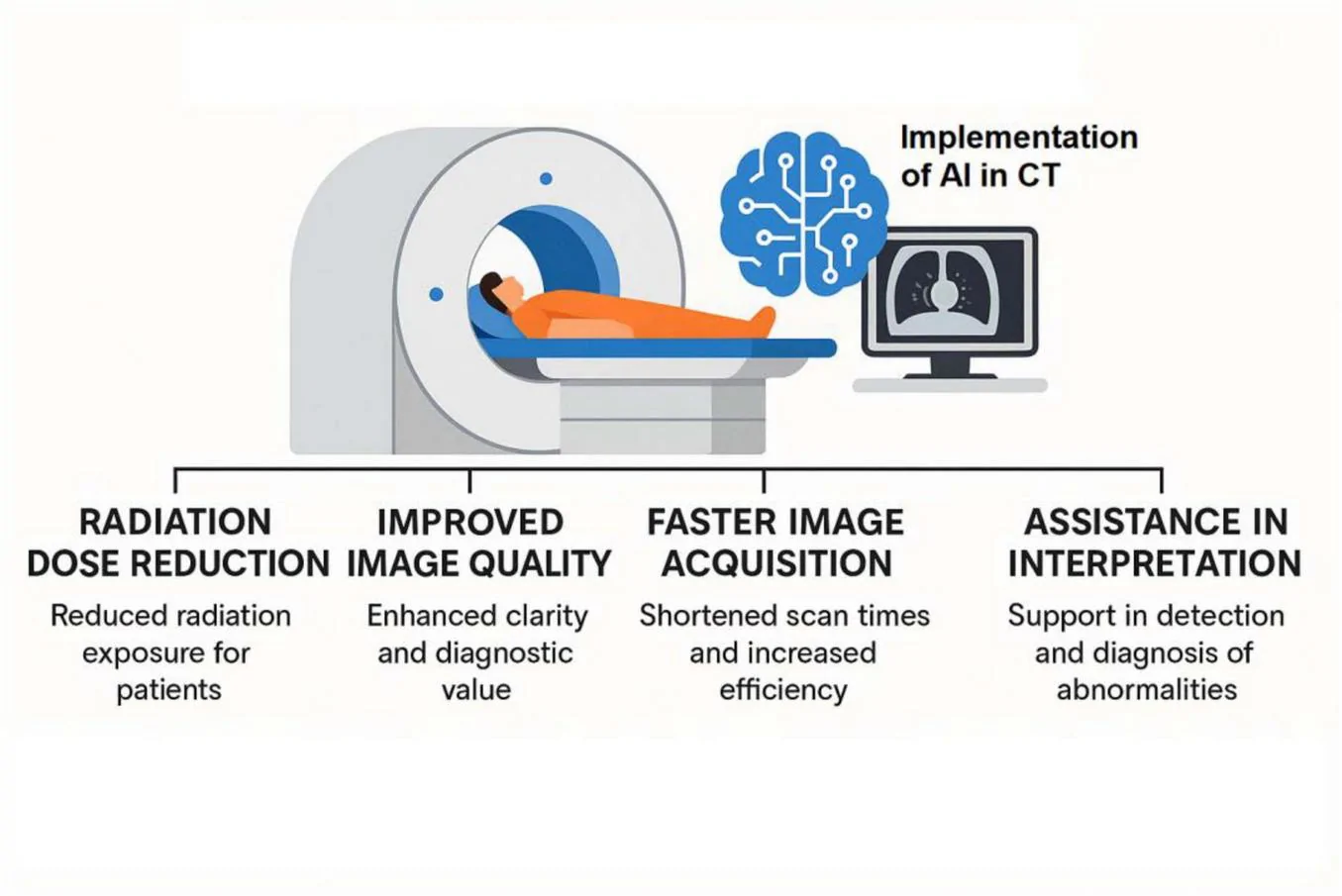
Implication of artificial intelligence (AI) in computed tomography (CT).

Artificial intelligence has great potential in advancing personalized CT dose optimization, offering more efficient, precise, and safer diagnostic imaging. By addressing the challenges mentioned above, future research on AI can redefine radiology practices across diverse healthcare settings, improving patient outcomes, reducing radiation risks, and ensuring diagnostic accuracy and data privacy (44, 45). Furthermore, collaboration between researchers, clinicians, and technology developers should be an important step to refine AI models and create standardized guidelines for their implementation (Figure 5).

## Data Availability

The original contributions presented in this study are included in this article/supplementary material, further inquiries can be directed to the corresponding author.
